# Efficacy and safety of moxifloxacin in acute exacerbations of chronic bronchitis: a prospective, multicenter, observational study (AVANTI)

**DOI:** 10.1186/1471-2466-13-5

**Published:** 2013-01-23

**Authors:** Alexander Chuchalin, Maryna Zakharova, Dejan Dokic, Mahir Tokić, Hans-Peter Marschall, Thomas Petri

**Affiliations:** 1 Pulmonology Department, Federal State Institution “Research Institute of Pulmonology of Roszdrav”, Moscow, 105077, Russian Federation; 2 Pulmonology Department, Hospital #7, Simferopol, 95044, Ukraine; 3 Klinika za Pulmologija i Alergologija, Skopje, 1000, Macedonia; 4 Privatna Pulmološka Ordinacija “Dr. Tokić”, 71 000, Sarajevo, Bosnia and Herzegovina; 5 Bayer Vital GmbH, Leverkusen, 51368, Germany; 6 Bayer Pharma AG, Berlin, 13353, Germany

**Keywords:** Antibiotics, Chronic bronchitis, COPD, Exacerbations, Moxifloxacin

## Abstract

**Background:**

Acute exacerbations of chronic bronchitis (AECB), including chronic obstructive pulmonary disease (AECOPD), represent a substantial patient burden. Few data exist on outpatient antibiotic management for AECB/AECOPD in Eastern/South Eastern Europe, in particular on the use of moxifloxacin (Avelox®), although moxifloxacin is widely approved in this region based on evidence from international clinical studies.

**Methods:**

AVANTI (AVelox® in Acute Exacerbations of chroNic bronchiTIs) was a prospective, observational study conducted in eight Eastern European countries in patients > 35 years with AECB/AECOPD to whom moxifloxacin was prescribed. In addition to safety and efficacy outcomes, data on risk factors and the impact of exacerbation on daily life were collected.

**Results:**

In the efficacy population (N = 2536), chronic bronchitis had been prevalent for > 10 years in 31.4% of patients and 66.0% of patients had concomitant COPD. Almost half the patients had never smoked, in contrast to data from Western Europe and the USA, where only one-quarter of COPD patients are non-smokers. The mean number of exacerbations in the last 12 months was 2.7 and 26.3% of patients had been hospitalized at least once for exacerbation. Physician compliance with the recommended moxifloxacin dose (400 mg once daily) was 99.6%. The mean duration of moxifloxacin therapy for the current exacerbation (Anthonisen type I or II in 83.1%; predominantly type I) was 6.4 ± 1.9 days. Symptom improvement was reported after a mean of 3.4 ± 1.4 days. After 5 days, 93.2% of patients reported improvement and, in total, 93.5% of patients were symptom-free after 10 days. In the safety population (N = 2672), 57 (2.3%) patients had treatment-emergent adverse events (TEAEs) and 4 (0.15%) had serious TEAEs; no deaths occurred. These results are in line with the known safety profile of moxifloxacin.

**Conclusions:**

A significant number of patients in this observational study had risk factors for poor outcome, justifying use of moxifloxacin. The safety profile of moxifloxacin and its value as an antibiotic treatment were confirmed. Physicians complied with the recommended 400 mg once-daily dose in a large proportion of patients, confirming the advantages of this simple dosing regimen.

**Trial registration:**

ClinicalTrials.gov identifier: NCT00846911

## Background

Acute exacerbations of chronic bronchitis (AECB), including chronic obstructive pulmonary disease (AECOPD), represent a substantial disease burden to patients, contributing to reduced lung function, increased morbidity and mortality, and long-term impairment in quality of life [1-8].

A role for bacteria is implicated in 40-50% of AECB episodes [9]. In a routine clinical setting, where bacteriological assessment may not be available, empirical antibacterial therapy is generally recommended for patients who fulfill specific clinical criteria, with the aim to influence the disease course and prevent complications [10-12]. Guidelines by Woodhead et al. [12] recommend antibiotic therapy for patients with increased dyspnea, sputum volume, and sputum purulence (Anthonisen type I) and for patients with two of these symptoms including increased sputum purulence (Anthonisen type II), but not in patients with one of these symptoms alone (Anthonisen type III) [13]. The GOLD recommendations for antibiotic therapy are based on the severity of exacerbations, the presence of risk factors, and predictors of poor outcome (e.g. comorbid conditions, frequency of AECBs, and previous antibiotic use) [10]. Using these criteria, the GOLD guidelines recommend amoxicillin/clavulanate or fluoroquinolones in patients with moderate to severe exacerbations.

Moxifloxacin is a fourth-generation fluoroquinolone with a broad spectrum of activity relevant to the microorganisms isolated in AECB, including Gram-positive and Gram-negative bacteria, atypical pathogens, and anaerobic bacteria, as well as species resistant to aminoglycosides, tetracyclines, and macrolide antibiotics. Beta-lactamase producing strains of *Haemophilus influenzae* and *Moraxella catarrhalis* are susceptible to moxifloxacin [14-17]. Moxifloxacin is strongly targeted to alveolar tissue [18,19] and demonstrates rapid initial killing and eradication rates for pneumococcal bacteria [16].

The initial clinical program for moxifloxacin in AECB included two studies of moxifloxacin (400 mg once daily, 5 days) versus clarithromycin (500 mg twice daily, 7–10 days) and two studies versus cefuroxime axetil (500 mg twice daily, 10 days) in a total of 2381 patients [20,21]. Together, these studies demonstrated that moxifloxacin achieved a clinical response rate of 89% and a bacteriological response rate of 87% at 7–14 days post-treatment.

In another, prospective, multicenter, randomized, double-blind study of outpatients with AECB (MOSAIC), 5-day moxifloxacin was associated with significantly higher clinical cure rates and bacterial eradication rates than a 7-day standard regimen (i.e. amoxicillin 500 mg three times daily, or clarithromycin 500 mg twice daily, or cefuroxime axetil 250 mg twice daily) [22]. In addition, the time until next exacerbation was significantly greater with moxifloxacin than the comparator during 9-month follow-up [22], which may be attributed to more effective bacterial eradication by moxifloxacin [23]. *Post-hoc* analyses of the MOSAIC study identified a beneficial influence on clinical cure rates from moxifloxacin treatment and a poorer outcome associated with cardiopulmonary disease, forced expiratory volume in 1 second (FEV_1_) < 50% predicted, and ≥ 4 AECBs in the previous year [24].

The recent MAESTRAL study of 1492 outpatients aged ≥ 60 years with moderate-to-severe AECOPD (Anthonisen grade I) showed that moxifloxacin (400 mg/day for 5 days) is as effective as amoxicillin/clavulanic acid (875/125 mg for 7 days) in clinical success rate, with a significantly lower failure rate in patients with confirmed bacterial AECOPD [25]. The benefits of moxifloxacin (400 mg/day for 5 days) also translated into a more favorable long-term quality of life when compared with amoxicillin/clavulanate (500/125 mg three times daily for 10 days) in the general practice setting [26].

Based on the existing controlled trial evidence, researchers have concluded that moxifloxacin is as effective or even more effective compared with other antimicrobials, with a more advantageous dosage regimen that may be associated with increased compliance [27,28].

Observational studies provide valuable information, alongside controlled clinical studies, with relevance to contemporary practice. Published data on 9225 patients aged ≥ 35 years with AECB or AECOPD from eight European countries, from among the 46 893 patients recruited globally to an observational study of moxifloxacin (the GIANT study), demonstrated very good or good efficacy for moxifloxacin in 94.9% of patients and very good or good tolerability in 96.7%, based on physician assessments [29].

Few data exist on the outpatient antibiotic management of AECB/AECOPD in Eastern/South Eastern Europe, and in particular on the use of moxifloxacin in this population. The current non-interventional observational study was conducted to gain further information on the treatment of AECB with moxifloxacin in a large population of outpatients with moderate-to-severe AECB recruited from countries in South Eastern/Eastern Europe and Kazakhstan.

## Methods

### Study design

The AVANTI study (AVelox® in Acute Exacerbations of chroNic bronchiTIs) was a prospective, multicenter, observational study conducted at 182 investigational centers in 8 countries (Albania, Bosnia and Herzegovina, Kazakhstan, Macedonia, Moldova, Russian Federation, Slovakia, and Ukraine) between 8 April 2008 and 6 April 2010.

The observational period for each patient commenced at the initiation of treatment with moxifloxacin (Avelox®) for AECB and was continued until an improvement or relief of symptoms at follow-up visit or premature discontinuation. Up to two follow-up visits were planned, with the last assessment following the final intake of moxifloxacin.

### Patients

Male or female outpatients aged ≥ 35 years with a diagnosis of AECB were included in the study. The diagnosis of AECB and of any concomitant diseases was provided by attending physicians, who were pulmonologists or internal medicine specialists (approximately 60%), general practitioners (10%), or practitioners from other specialties. An exacerbation of chronic bronchitis was considered to be present when the patient experienced an acute increase in respiratory symptoms, including dyspnea, sputum volume, and/or sputum clearance. Exacerbations were classified into Anthonisen types I, II, or III [13]. Exclusion criteria were limited to contraindications to the use of moxifloxacin, as described in the locally available Summary of Product Characteristics.

Data on disease characteristics, risk factors, and the impact of exacerbations on daily life were collected from patients before initiation of moxifloxacin treatment.

The study protocol was approved by the local independent ethics committee or institutional review board, as applicable, at each of the investigator sites. At the national level, the study was approved in Albania by the Bioethics National Committee of the Ministry of Health, in Moldova by the National Ethical Committee, in the Russian Republic by the Ethical Committee at the Federal Service on Surveillance in Healthcare and Social Development, in Slovakia by the Ethical Committee of the Bratislava Region, and in Ukraine by the Central Ethics Commission of the Ministry of Health. Notification on the study protocol, following regulatory requirements for non-interventional studies, was provided in Bosnia and Herzegovina to the Ministry of Health, and in Kazakhstan to the local regulatory authority and the National Center for Drug Expertise, Medical Devices and Medical Equipment. In Macedonia, no ethics committee approval or notification was requested at national level. All patients provided informed consent in accordance with local regulations.

### Study medication

Moxifloxacin was prescribed according to the medical judgment of the investigator and in accordance with the guidelines from the European Medicines Agency, the US Food and Drug Administration, and local regulations (e.g. Avelox® (moxifloxacin hydrochloride) US prescribing information [30]).

The dose of moxifloxacin recommended for the treatment of AECB in the study was 400 mg once daily, consistent with the local Summary of Product Characteristics. Final decisions on the dose of moxifloxacin and on the use of concomitant medications were at the discretion of the attending physician.

### Efficacy and safety assessments

Efficacy assessments for each patient included the frequency of improvement of different symptoms (including sputum volume, sputum character, fever, cough, and dyspnea), the frequency of cure (i.e. symptom-free status), the time to improvement in symptoms and to cure, and general assessments of the effectiveness of moxifloxacin treatment using methodologies similar to those employed in the GIANT study [29].

Safety evaluations included adverse events reported during the study, coded using the Medical Dictionary for Regulatory Activities (MedDRA) version 13.0, and a general subjective tolerability assessment by investigators.

Physicians additionally provided summary assessments of the overall efficacy and tolerability of moxifloxacin into the categories: ‘very good’, ‘good’, ‘sufficient’, and ‘insufficient’. Both physicians and patients provided an assessment of their satisfaction with the therapeutic effect of moxifloxacin. Finally, for patients with available data, physicians compared the overall effect and onset of action of moxifloxacin against the antibiotic used to treat the previous episode of AECB.

### Statistical analyses

Efficacy and safety outcomes were analyzed by descriptive statistics. As appropriate for non-interventional studies, statistical tests were not performed. The safety population included all patients who took at least one dose of study medication and provided information on adverse events. The efficacy population included all patients who took at least one dose of study medication and provided information on the efficacy of treatment.

A minimum of 1600 patients were planned to be included in the study. As 2672 patients were actually included, adverse events occurring at a frequency of 0.125% (1:800 patients) could be detected with a probability of 95%.

## Results

### Patient population

**Figure 1:**
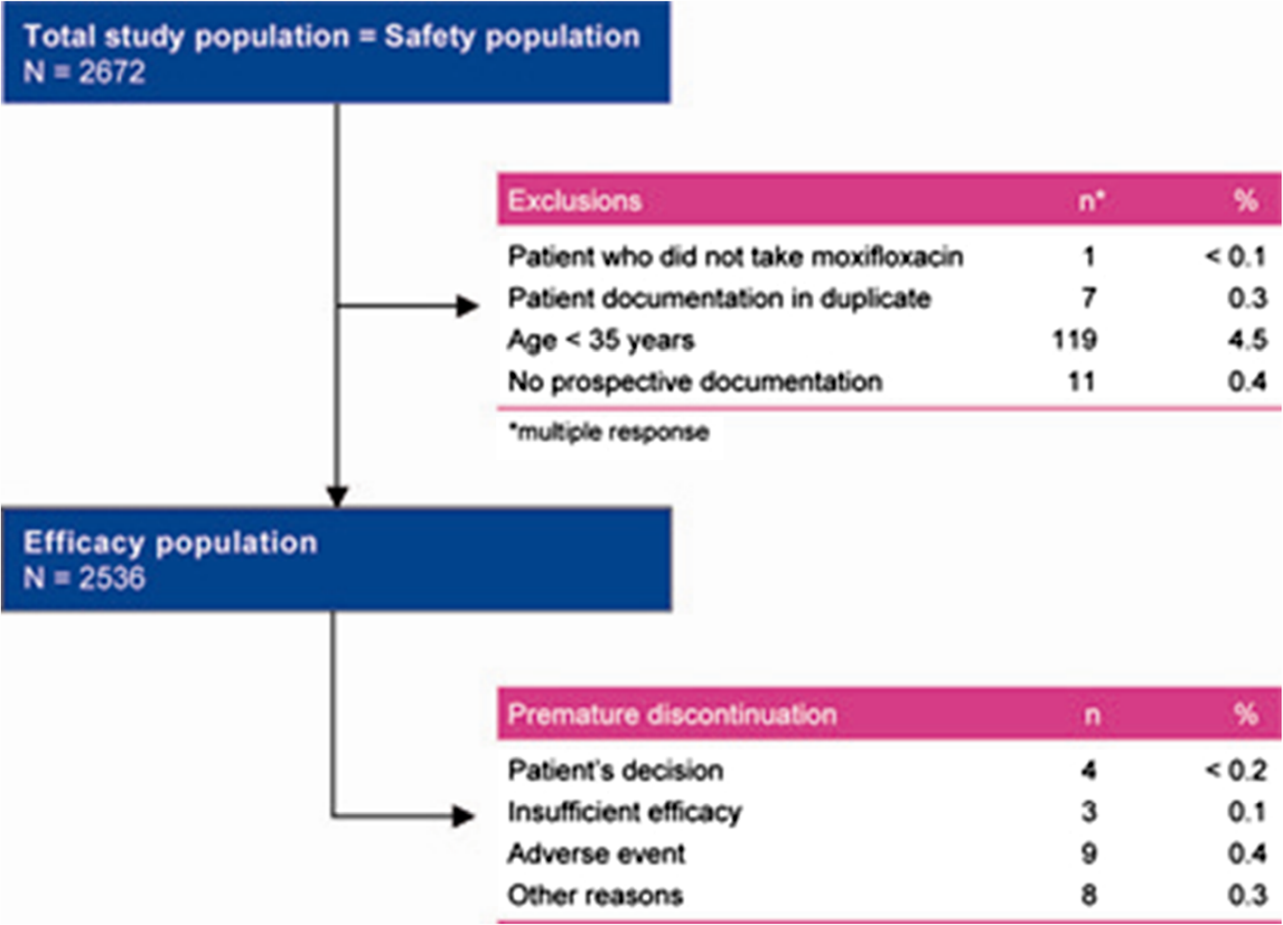
Patient disposition.

A total of 2672 patients were enrolled in the study and included in the safety population. The efficacy population consisted of 2536 patients, after exclusion of 136 (5.1%) patients from the safety population, most commonly because of age < 35 years (n = 119) (Figure 1).

Demographic and disease characteristics of the efficacy population at baseline are presented in Table 1. Patient ages ranged from 35 to 94 years, with approximately one-third of patients (31.6%) aged above 65 years. Over one-half of patients (53.9%) were past or current smokers. Over the past 12 months, patients had experienced a mean of 2.7 ± 1.9 (range 1.0-20.0) episodes of AECB. An antibiotic was prescribed for the previous episode of AECB in 62.2% of patients, most commonly amoxicillin (13.6% of patients), usually combined with clavulanic acid.

**Table 1:** Patient demographics and disease characteristics at baseline (efficacy population)

**Parameter**	**Total N = 2536 (100%)**
Gender, n (%)	
Male	1441 (56.8)
Female	1095 (43.2)
Mean (SD) age, y (n = 2532)	57.8 (12.2)
Mean (SD) weight, kg (n = 2504)	78.8 (15.3)
Mean (SD) height, cm (n = 2477)	170.4 (8.5)
Mean (SD) BMI, kg/m^2^ (n = 2477)	27.1 (4.8)
Race, n (%)	
White	2349 (92.6)
Asian	73 (2.9)
Black	7 (0.3)
Other	6 (0.2)
Missing	101 (4.0)
Frequency of common symptoms, n (%)	
Sputum purulence increased	2021 (79.7)
Worsening dyspnea	1998 (78.8)
Sputum volume increased	1707 (67.3)
Upper respiratory tract infection (past 5 days)	1266 (49.9)
Anthonisen grade, n (%)	
Type I	1089 (42.9)
Type II	1019 (40.2)
Type III	412 (16.2)
Missing	16 (0.6)
Smoking status, n (%)	
Past or current	1367 (53.9)
Never	1159 (45.7)
Missing	10 (0.4)
Years with chronic bronchitis, n (%)	
< 1	127 (5.0)
1-5	863 (34.0)
> 5-10	748 (29.5)
> 10	796 (31.4)
Missing	2 (0.1)
Exacerbations in past 12 months	
None	478 (18.8)
Yes	2048 (80.8)
1	479 (18.9)
2	745 (29.4)
3	420 (16.6)
4	198 (7.8)
5	97 (3.8)
≥ 6	109 (4.3)
Missing	10 (0.4)
Hospitalization due to AECB in past 12 months	
None	1867 (73.6)
Yes	668 (26.3)
1	442 (17.4)
2	164 (6.5)
3	35 (1.4)
4	10 (0.4)
≥ 5	17 (0.7)
Missing	1 (< 0.1)
Corticosteroid intake in past 12 months	
Yes	964 (38.0)
No	1571 (61.9)
Missing	1 (< 0.1)
Antibiotic treatment for last AECB	
Yes	1577 (62.2)
No	959 (37.8)
Missing	0 (0.0)

AECB, acute exacerbation of chronic bronchitis.

The most common symptoms in the current AECB episode were increased sputum purulence, worsening of dyspnea, and increased sputum volume (Table 1); 42.9% of patients were classified as Anthonisen type I, 40.2% as type II, and 16.2% as type III, with data missing in the remainder (Table 1). One-half of patients (49.9%) complained of an infection of the upper respiratory tract in the past 5 days. An impact on daily life activities was reported by 90.4%, over a mean duration of 6.6 ± 5.5 days. Sleep disturbances were reported by 68.6% of patients, with impact on a mean of 4.1 ± 3.7 nights. The impact of the current AECB episode on daily activities and sleep disturbance in patient subgroups categorized by gender, age, smoking status, concomitant diseases, Anthonisen grade, and number of severe symptoms is presented in Table 2. Mean FEV_1_ (measured in 1261 patients) was 2.0 ± 0.9 liters. Patients experienced AECB symptoms for a mean of 7.0 ± 5.0 days before initiation of treatment with moxifloxacin.

**Table 2:** Impact of current AECB episode on daily life activities and sleep disturbance (efficacy population)

**Parameter**	**Days with impact on daily life activities**			**Nights with sleep disturbance**		
	**(N = 2292)**			**(N = 1740)**		
	**n (%)**	**Mean**	**SD**	**n (%)**	**Mean**	**SD**
**Gender**						
Male	1308 (57.1)	6.6	5.3	971 (55.8)	4.1	3.8
Female	984 (42.9)	6.6	5.8	769 (44.2)	4.1	3.6
Missing	0 (0)	-	-	0 (0)	-	-
**Age group (years)**						
≥ 35 to < 50	615 (26.8)	6.4	5.2	406 (23.3)	3.7	2.7
≥ 50 to < 65	922 (40.2)	6.7	5.9	712 (40.9)	4.0	4.1
≥ 65 to < 80	680 (29.7)	6.7	5.3	557 (32.0)	4.5	3.8
≥ 80	71 (3.1)	5.8	3.9	63 (3.6)	4.3	3.0
Missing	4 (0.2)	11.8	5.2	2 (0.1)	4.0	1.4
**Smoking status**						
Never	1027 (44.8)	6.7	5.6	786 (45.2)	4.0	3.7
Past or current smoker	1255 (54.8)	6.5	5.4	945 (54.3)	4.2	3.7
Missing	10 (0.4)	8.7	5.3	9 (0.5)	3.6	2.1
**Concomitant diseases of special interest**						
COPD	1549 (67.6)	6.6	5.6	1216 (69.9)	4.3	3.9
Asthma	389 (17.0)	7.5	6.8	331 (19.0)	4.4	3.5
Emphysema	568 (24.8)	7.7	6.4	461 (26.5)	4.5	3.7
Bronchiectasis	151 (6.6)	7.3	6.9	128 (7.4)	4.6	4.7
Cor pulmonale	251 (11.0)	8.1	6.4	222 (12.8)	5.1	4.2
Cardiomyopathy	146 (6.4)	7.9	7.4	122 (7.0)	5.4	5.0
Cardiac ischemia	544 (23.7)	7.2	6.3	429 (24.7)	4.2	3.5
Heart insufficiency	135 (5.9)	10.9	10.1	126 (7.2)	6.4	6.3
Cardiac arrhythmia	145 (6.3)	7.7	5.1	131 (7.5)	4.9	4.0
Chronic alcoholism	39 (1.7)	6.0	6.8	24 (1.4)	5.3	5.5
Diabetes	238 (10.4)	6.3	5.3	196 (11.3)	4.1	3.2
No diseases of special interest	324 (14.1)	5.8	4.1	203 (11.7)	3.0	2.8
**Anthonisen grade**						
Type I	1014 (44.2)	7.1	5.1	793 (45.6)	4.5	3.8
Type II	913 (39.8)	6.5	5.9	666 (38.3)	3.8	3.6
Type III	353 (15.4)	5.6	5.6	274 (15.7)	3.6	3.3
Missing	12 (0.5)	7.4	6.2	7 (0.4)	7.3	7.5
**Number of severe symptoms per patient at start of therapy**						
1	446 (19.5)	6.0	5.2	295 (17.0)	3.5	3.5
2	596 (26.0)	6.0	5.0	417 (24.0)	3.5	3.3
3	377 (16.4)	6.3	5.0	279 (16.0)	4.1	3.1
4	241 (10.5)	6.8	5.4	193 (11.1)	4.4	3.9
5	163 (7.1)	7.7	4.9	142 (8.2)	4.9	3.4
6	142 (6.2)	10.2	7.9	141 (8.1)	5.8	3.7
7	66 (2.9)	9.6	7.5	66 (3.8)	6.2	5.8
8	16 (0.7)	10.4	9.6	15 (0.9)	8.1	8.6
None	245 (10.7)	5.6	4.6	192 (11.0)	3.2	3.1

AECB, acute exacerbation of chronic bronchitis; COPD, chronic obstructive pulmonary disease.

Concomitant diseases of special interest recorded by investigators included COPD (66.0% of patients), emphysema (23.8%), asthma (16.6%), cardiac ischemia (23.1%), cor pulmonale (10.6%), and diabetes (10.1%). Concomitant medications were taken by 93.3% of patients, most commonly a corticosteroid (32.8% overall, including 40.8% of Anthonisen type I, 28.0% of type II, and 24.2% of type III patients); the mucolytic ambroxol (18.8%); and the mucolytic/antioxidant, acetylcysteine (18.6%). As expected, a large proportion of patients (83.4%) received comedications to treat their respiratory symptoms.

The most frequently used non-AECB-related comedications were for the treatment of cardiovascular symptoms (36.1% of patients), dermatological diseases (23.0%), dysfunction of the alimentary tract and metabolism (22.2%), and ophthalmological diseases (20.5%).

### Moxifloxacin treatment

Moxifloxacin was administered at the recommended dose of 400 mg once daily in 99.6% (n = 2526) of enrolled patients, with a higher dose of 600 mg/day (n = 5) or 800 mg/day (n = 5) in the remainder.

The mean (SD) duration of moxifloxacin treatment was 6.4 ± 1.9 days (range: 1.0-15.0 days) in the efficacy population; 55.2% of patients were treated for 5 days, 29.1% for 7 days, and 14.0% for 10 days. Mean durations of treatment in patients with Anthonisen type I, type II, and type III AECB were 6.4 ± 1.9, 6.4 ± 1.9, and 6.2 ± 1.8 days, respectively, and were 6.3 ± 1.9 days in never smokers versus 6.5 ± 1.9 days in past or current smokers. Durations of treatment were 6.0 ± 1.8 days for patients aged < 50 years, 6.4 ± 1.9 for age ≥ 50 to < 65, 6.7 ± 2.0 days for age ≥ 65 to < 80, and 7.0 ± 1.9 days for age ≥ 80 years.

The last follow-up visit was performed after a mean of 9.8 ± 6.2 days (range 2–66 days) from the initiation of moxifloxacin treatment. Moxifloxacin was discontinued prematurely in 23 (0.9%) patients, because of the patient’s decision (n = 4), insufficient efficacy (n = 3), adverse events (n = 9), and ‘other reasons’ (n = 8) (multiple responses included).

### Efficacy assessments of moxifloxacin treatment

An improvement or relief of the symptoms of AECB that were present at baseline was reported in 89.4% of patients for sputum volume, 97.2% for fever, 86.0% for cough, 87.7% for dyspnea, and 77.2% for sputum character during moxifloxacin treatment. Additional symptom changes are presented in Table 3.

**Table 3:** Course of symptoms during observational period; patients with symptoms at initial visit (efficacy population)

**Symptom**	**Total**	**Relieved**	**Improved**	**Unchanged**	**Worsened**	**Missing**
	**n (%)**^**a**^	**n (%)**^**b**^	**n (%)**^**b**^	**n (%)**^**b**^	**n (%)**^**b**^	**n (%)**^**b**^
**Fever**	1768 (69.7)	1713 (96.9)	5 (0.3)	15 (0.8)	0 (0.0)	35 (2.0)
**Cough**	2512 (99.1)	1666 (66.3)	495 (19.7)	320 (12.7)	1 (< 0.1)	30 (1.2)
**Dyspnea**	2298 (90.6)	1615 (70.3)	399 (17.4)	245 (10.7)	1 (< 0.1)	38 (1.7)
**Sputum volume**	2471 (97.4)	1364 (55.2)	846 (34.2)	226 (9.1)	8 (0.3)	27 (1.1)
**Sputum character**	2284 (90.1)	1350 (59.1)	446 (19.5)	73 (3.2)	1 (< 0.1)	414 (18.1)
**Chest discomfort**	2116 (83.4)	1822 (86.1)	103 (4.9)	152 (7.2)	1 (< 0.1)	38 (1.8)
**Fatigue**	1984 (78.2)	1573 (79.3)	180 (9.1)	186 (9.4)	0 (0.0)	45 (2.3)
**Sleep disturbances**	1672 (65.9)	1521 (91.0)	45 (2.7)	78 (4.7)	1 (0.1)	27 (1.6)

^a^Proportion of the efficacy population (n = 2536); ^b^proportion of the patients who had symptoms at initial visit.

Improvement in symptoms occurred after a mean of 3.4 ± 1.4 days of moxifloxacin treatment. Improvements occurred by 3 days in 60.7% of patients, 5 days in 93.2%, and 10 days in 99.3%. Only 0.6% of patients (n = 14) experienced no symptom improvements during the observational period.

The mean duration of treatment until symptom improvement in patients with Anthonisen type I, II, and III AECB was 3.6 ± 1.5, 3.3 ± 1.4, and 3.4 ± 1.4 days, respectively (Table 4), 3.4 ± 1.4 days in never smokers versus 3.5 ± 1.5 days in past or current smokers, 3.6 ± 1.6 days in concomitant corticosteroid users versus 3.4 ± 1.3 in non-corticosteroid users, and 3.7 ± 1.5 days in patients with >3 exacerbations versus 3.4 ± 1.4 days in patients with ≤3 exacerbations in the previous 12 months. Duration of treatment until symptom improvement was 3.2 ± 1.4 days in patients aged < 50 years, 3.4 ± 1.4 days for age ≥ 50 to < 65, 3.6 ± 1.5 days for age ≥ 65 to < 80, and 3.7 ± 1.9 for age ≥ 80 years. Mean duration of treatment until symptom improvement in patients with concomitant diseases is described in Table 4. For patients with COPD (diagnosed by the attending physician), the mean duration of moxifloxacin treatment until improvement was 3.5 ± 1.4 days.

**Table 4:** Duration of treatment until symptom improvement (efficacy population)

**Parameter**	**Duration until improvement**		
	**n**	**Mean**	**SD**
**Anthonisen grade**			
Type I	1084	3.6	(1.5)
Type II	1011	3.3	(1.4)
Type III	409	3.4	(1.4)
Missing	16	4.2	(1.6)
**Concomitant diseases of special interest**			
COPD	1667	3.5	1.4
Asthma	412	3.4	1.4
Emphysema	602	3.6	1.5
Bronchiectasis	162	3.8	1.4
Cor pulmonale	268	3.6	1.4
Cardiomyopathy	157	3.5	1.5
Cardiac ischemia	582	3.7	1.6
Heart insufficiency	138	3.1	1.3
Cardiac arrhythmia	151	3.7	1.6
Diabetes	255	3.6	1.7

COPD, chronic obstructive pulmonary disease.

**Figure 2:**
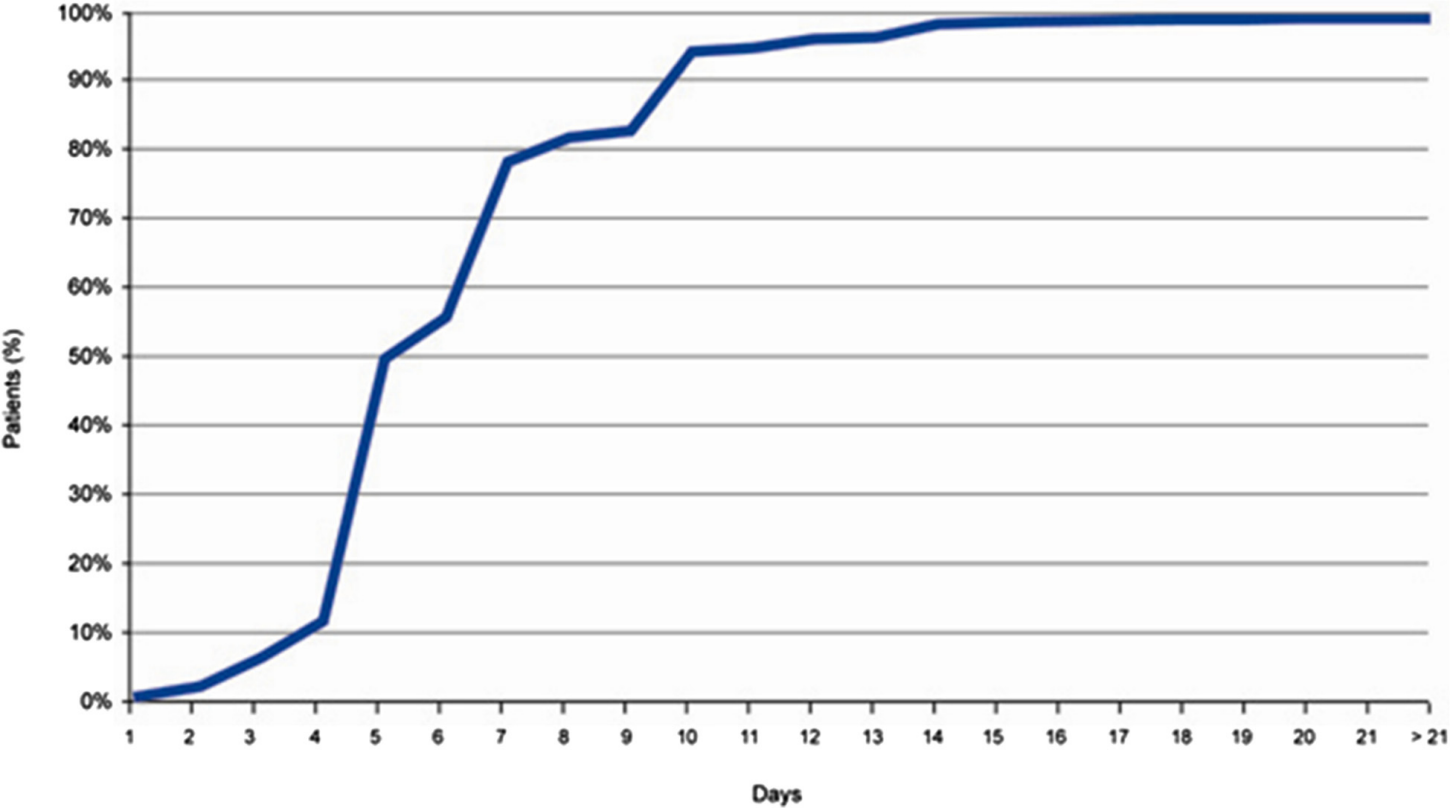
Cumulative increase in proportion of symptom-free patients during moxifloxacin treatment.

The mean duration until attainment of a symptom-free status was 6.5 ± 2.7 days. A total of 49.1% of patients were symptom-free after 5 days, 77.6% after 7 days, 93.5% after 10 days, and 98.3% after 20 days (Figure 2). Only 1.4% of observed patients (n = 36) were reported not to attain symptom-free status during the observational period.

### Safety assessments

Treatment-emergent adverse events (TEAEs) were reported in 2.13% (n = 57) patients during the observational period. The most common TEAEs included diarrhea (0.52%, n = 14 patients), nausea (0.41%, n = 11), dizziness (0.30%, n = 8), dyspepsia (0.22%, n = 6), fatigue (0.15%, n = 4), and headache (0.15%, n = 4). TEAEs considered potentially drug related were reported in 1.91% (n = 51) patients. Moxifloxacin treatment was interrupted in eight of these patients, withdrawn in four, and the dose was reduced in one patient. By the end of the observational period, drug-related TEAEs had resolved in 40 of the 51 patients, resolved with sequelae in another three, and improved in eight patients.

Four patients (0.15%) experienced 11 serious TEAEs (n = 2, atrial fibrillation; n = 1 each of: acute myocardial infarction, cardiac flutter, diplopia, vomiting, allergic edema, amnesia, dizziness, dyspnea, and skin reaction). The serious TEAEs were considered to be drug related. All serious TEAEs had resolved by the end of the observational period, following interruption of moxifloxacin treatment in three patients and treatment withdrawal in one patient.

None of the 10 patients who received moxifloxacin at above the recommended dose of 400 mg once daily (n = 5, 600 mg/day; n = 5, 800 mg/day) experienced an adverse event.

### Summary assessments of moxifloxacin treatment

The efficacy of moxifloxacin was rated by physicians as ‘very good’ or ‘good’ in 97.7% of patients, ‘sufficient’ in 1.8%, and ‘insufficient’ in 0.5%. Physicians’ assessments of the efficacy of moxifloxacin in patient subgroups categorized by gender, age, and Anthonisen grade are presented in Table 5. The tolerability of moxifloxacin was rated by physicians as ‘very good’ or ‘good’ in 97.8% of patients, ‘sufficient’ in 1.8%, and ‘insufficient’ in 0.3%.

**Table 5:** Physician’s assessments of efficacy of moxifloxacin (efficacy population)

**Parameter**	**Total**	**Very good / good**	**Sufficient**	**Insufficient**
	**n**	**n (%)**	**n (%)**	**n (%)**
**Gender**				
Male	1441	1405 (97.5)	30 (2.1)	6 (0.4)
Female	1095	1072 (97.9)	15 (1.4)	7 (0.6)
Missing	0	0 (0)	0 (0)	0 (0)
**Age group (years, n = 2462**)				
≥ 35 to < 50	707	694 (98.2)	9 (1.3)	4 (0.6)
≥ 50 to < 65	1023	999 (97.7)	20 (2.0)	4 (0.4)
≥ 65 to < 80	728	707 (97.1)	15 (2.1)	5 (0.7)
Missing	4	4 (100)	0 (0)	0 (0)
**Anthonisen grade**				
Type I	1089	1065 (97.8)	17 (1.6)	7 (0.6)
Type II	1019	995 (97.6)	21 (2.1)	3 (0.3)
Type III	412	401 (97.3)	7 (1.7)	3 (0.7)
Missing	16	16 (100)	0 (0)	0 (0)

Approximately 99% of both physicians and patients stated that they were ‘very satisfied’ or ‘satisfied’ with the therapeutic effect of moxifloxacin. Compared with the antibiotic treatment during the previous episode of AECB, physicians rated moxifloxacin as better in 77.5% of patients, equal in 5.3%, and worse in 0.2%, with missing data in 17.0%. Moxifloxacin was considered to have an earlier onset of action compared with the previous antibiotic in 73.5% of patients, equivalent onset in 9.5%, and later onset in 1.3%, with missing data in 15.7%. Physicians reported that they would prescribe moxifloxacin again in 98.1% of patients.

## Discussion

This non-interventional, naturalistic observational study enrolled a large cohort of outpatients (n = 2672) with AECB, Anthonisen types I to III, to receive moxifloxacin treatment at the recommended dose of 400 mg once daily. A special feature of the study is the well-documented patient history regarding previous AECBs, concomitant diseases, and comedications related both to the underlying respiratory disease as well as to other comorbidities before study entry. The majority of patients (approximately 80%) had experienced an exacerbation within the previous 12 months. Also reflecting current clinical experience, a large proportion of the patients had an underlying respiratory condition (e.g. COPD, emphysema, or asthma).

Moxifloxacin administered for a mean of 6.4 days (range 1–15 days) was a highly effective treatment in these patients. Individual symptoms and signs of sputum volume, fever, cough, dyspnea, and sputum character resolved or improved in the majority of patients (range 77-89%) during the observational period. Improvements in symptoms occurred after a mean of 3.4 days and over 93% of patients were symptom-free after 10 days. No differences in the efficacy of moxifloxacin were observed between patients either without or with a diverse range of comorbidities.

Unlike in clinical trials, the dosing regimen used in this non-interventional study was left to the sole discretion of the treating physician. It is interesting to note the high rate of physician compliance (99.6%) with the dose recommended in the Summary of Product Characteristics. This suggests that physicians considered the recommended dose of moxifloxacin to be highly effective, without the need to adjust the dose, e.g. for body weight. The lack of need for dose adjustment has the advantages of easier dosing and a reduced risk of overdosing.

The results of this study are in agreement with previous studies of moxifloxacin treatment in patients with AECB, including the international observational GIANT study, where symptom improvement occurred after a mean of 3.4 days [29]. Physicians’ summary assessments of moxifloxacin were also similar in the two studies, including a rating of ‘very good or good’ in excess of 95% of patients.

The rapid recovery from symptoms observed in this study is a desirable characteristic of an effective treatment for patients with AECB. Other observational and controlled studies and cross-sectional analyses report that moxifloxacin is associated with a more rapid recovery from symptoms than other commonly used treatments [31-33]. The mean duration of treatment until symptom improvement in the current study was broadly similar among patients, but with a trend to increased treatment duration in patients with greater AECB severity, concomitant diseases, and older age.

Physicians rated the tolerability of moxifloxacin as ‘very good or good’ in approximately 98% of patients, similar to the rate (97%) reported in the observational study by Miravitlles et al. [29]. Incidences of TEAEs and drug-related adverse events were low. The incidence of TEAEs was lower in the current study than reported in controlled clinical studies (e.g. [34]), which may be attributed to an underreporting of mild/moderate adverse events that is a feature of observational studies.

The profile of adverse events reported in this study is in agreement with current knowledge of this antibiotic [25,29,35,36]. A meta-analysis of clinical trial and post-marketing surveillance data for moxifloxacin identified nausea, dizziness, and diarrhea as the most frequent adverse events, which occurred at a rate similar to comparator medications [35]. For most patients in the current study, adverse events resolved during the course of treatment and were associated with low rates of treatment withdrawal (0.4%). The observational study by Miravitlles et al. [29] reported similarly low rates of treatment-related withdrawal (0.6%).

The overall satisfaction with moxifloxacin treatment expressed by both physicians and patients was high. Relative to previous antibiotics, moxifloxacin also provided a superior efficacy and a faster onset of effect in the majority of patients.

Notable demographic and disease characteristics of this population from South Eastern/Eastern Europe include a markedly higher incidence of COPD among non-smokers when compared with data from Western Europe and the USA [37-39]. This indicates that additional environmental factors, such as high levels of industrial air pollution and/or occupational or home indoor air pollution, contributed to the development of COPD in patients from the participating countries, as described by Mannino and Buist [40].

Limitations of the current study include the primary role of physician judgment for decisions on patient selection and management; the absence of a control group to quantify the response to other antibacterial agents; and the lack of bacteriological assessment, which precludes a correlation with the clinical outcomes. All prescribing choices were made by physicians. As approximately 16% of patients who received moxifloxacin were classified with Anthonisen type III AECB, antibiotic therapy was not prescribed in accordance with current guidelines in all circumstances. A similar experience was reported in the GIANT study [29].

A strength of observational studies is that they provide an important accompaniment to randomized controlled trials and reflect real-world practice in terms of prescribing behavior [41]. The lack of bacteriological assessment in this study is in line with current practice for the outpatient treatment of AECB. The high response rate in this study, which included patients with a range of common comorbidities, suggests that treatment with broader-spectrum drugs such as moxifloxacin is appropriate for patients with moderate-to-severe AECB who are managed outside hospital.

## Conclusions

The efficacy, safety, and tolerability profiles of moxifloxacin that are characterized in this large observational study from South Eastern/Eastern Europe confirm previous studies which report that moxifloxacin offers benefits for the treatment of moderate-to-severe exacerbations in outpatients with AECB. The response to moxifloxacin treatment was broadly independent of the patients’ demographic and disease background. Physicians complied with the recommended 400 mg once-daily dose in a large proportion of patients, confirming the advantages of this simple dosing regimen.

## Abbreviations

AECB: Acute exacerbation of chronic bronchitis; AECOPD: Acute exacerbation of chronic obstructive pulmonary disease; COPD: Chronic obstructive pulmonary disease; FEV_1_: Forced expiratory volume in 1 second.

## Competing interests

AC, MZ, DD, and MT declare that they have no competing interests.

H-PM and TP are full-time employees of Bayer AG.

## Authors’ contributions

AC participated in study design, data acquisition, and data analysis and interpretation. MZ, DD, MT, and H-PM participated in data acquisition and data analysis and interpretation. TP participated in study design and concepts, and data analysis and interpretation. All authors participated in the critical review revision of the manuscript. All authors read and approved the final manuscript for submission.

## Pre-publication history

The pre-publication history for this paper can be accessed here:

http://www.biomedcentral.com/1471-2466/13/5/prepub
